# Athletes with Implantable Cardioverter-Defibrillators: A Practical Framework for Device Selection, Programming, and Return-to-Sport Risk Mitigation

**DOI:** 10.3390/jcm15187103

**Published:** 2026-09-13

**Authors:** Demetrio Sharp Dimitri, Jordan M. Prutkin

**Affiliations:** Division of Cardiology, Department of Medicine, University of Washington, Seattle, WA 98195, USA; dsharpd@uw.edu

**Keywords:** implantable cardioverter-defibrillator, athlete, sports cardiology, electrophysiology, return to sport, device programming, subcutaneous ICD, extravascular ICD, shared decision-making, sudden cardiac death

## Abstract

Implantable cardioverter-defibrillators (ICDs) were historically viewed as a barrier to vigorous or competitive sport participation, based on theoretical concerns about arrhythmia induction, inappropriate shocks, device damage, and shock-related injury during activity. Contemporary registry data have reframed this paradigm, demonstrating that selected athletes with ICDs can participate in organized sport without the catastrophic outcomes once feared, although these data remain reassuring rather than definitive given selection bias and underrepresentation of high-consequence sports. This review synthesizes contemporary athlete-specific registry data and broader randomized device and programming evidence, alongside current guideline recommendations, to provide a practical framework for the care of athletes with ICDs, addressing indications, which should remain diagnosis-driven rather than sport-driven; device selection, weighing transvenous, subcutaneous, and extravascular platforms against an athlete’s pacing needs, anatomy, and sport-specific trauma risk; and exercise-informed device programming. We then present a longitudinal framework integrating clinical readiness, device readiness, sport-specific risk, emergency preparedness, shared decision-making, and reassessment after clinical or sporting changes. Special populations and priorities for future research are also discussed. Rather than a binary clearance decision, modern management of athletes with ICDs requires an individualized, longitudinally reassessed approach to risk.

## 1. Introduction

For decades, the presence of an implantable cardioverter-defibrillator (ICD) in an athlete was viewed as a barrier to vigorous or competitive sport participation. This cautious stance reflected concerns that intense exercise might increase the risk of ventricular arrhythmias, provoke inappropriate ICD shocks due to sinus tachycardia or supraventricular arrhythmias, damage the device or its leads, place the athlete at serious risk if syncope or a shock occurred during activity, or impair the ability of an ICD shock to terminate an arrhythmia. Consequently, earlier eligibility guidelines generally recommended restriction, particularly from competitive, high-intensity, collision, or other high-consequence sports [1,2,3,4].

Contemporary evidence has shifted this paradigm. Prospective registry data and longer-term follow-up studies have demonstrated that selected athletes with ICDs can participate in vigorous and competitive sports without the catastrophic outcomes that had previously been feared [5,6,7,8,9]. Reflecting these data, the 2025 AHA/ACC Clinical Considerations for Competitive Sports Participation for Athletes With Cardiovascular Abnormalities scientific statement considers competitive sports participation reasonable for athletes with an ICD after individualized risk assessment and shared decision-making. Participation in collision or impact sports can also be considered, provided that the discussion specifically addresses the potential for ICD-system damage or malfunction [10]. Importantly, an ICD mitigates the risk of malignant ventricular arrhythmia but does not eliminate the risk conferred by the underlying cardiac disease itself. Conditions such as arrhythmogenic cardiomyopathy, catecholaminergic polymorphic ventricular tachycardia, long QT syndrome, hypertrophic cardiomyopathy, and myocarditis each carry disease-specific considerations that persist independent of device protection and that inform return-to-sport decisions throughout this review.

These findings do not imply that sport participation is risk-free or appropriate for every ICD recipient. Rather, the clinical question has shifted from whether sport should be categorically prohibited to how clinicians can assess and mitigate risk through individualized counseling, device management, and return-to-sport planning.

In this review, we present a practical approach to caring for athletes with ICDs. We summarize the evidence that moved the field from broad restriction toward individualized return-to-sport decisions and integrate device selection, programming, sport-specific risk, emergency preparedness, and longitudinal reassessment. Our goal is to help clinicians support selected athletes in participating as safely as possible while acknowledging the uncertainties that should continue to inform counseling and follow-up.

Although the 2024 HRS consensus statement and the 2025 AHA/ACC scientific statement have substantially advanced guidance on ICD-specific return-to-sport decisions, both remain organized around broad, multi-condition eligibility recommendations rather than the device-level and workflow-level decisions clinicians face once an ICD is already indicated [9,10]. This review does not seek to supersede or duplicate these statements; rather, it translates their recommendations, together with device-selection and programming evidence not centrally addressed by either document, into an integrated, ICD-specific clinical workflow, and incorporates athlete-specific evidence published after these statements, including a 2025 meta-analysis of return-to-play outcomes [11], real-world subcutaneous ICD physical-activity data from the iSUSI registry [12], and recent case reports of exercise-guided subcutaneous ICD programming and extravascular ICD implantation in competitive athletes [13,14].

The 2024 HRS consensus statement and 2025 AHA/ACC scientific statement in the United States, and the 2020 ESC Sports Cardiology guidelines together with the 2022 ESC ventricular arrhythmia guideline in Europe, have converged on a broadly similar philosophy: shared decision-making has replaced categorical restriction as the governing principle for athletes with ICDs [4,9,10,15]. Some differences in emphasis remain. US documents typically stratify participation as competitive versus recreational, whereas the ESC guidelines add an explicit leisure-time category and, in ICD-specific decisions, direct clinicians to weigh explicitly that intensive sport increases the frequency of both appropriate and inappropriate shocks and the potential risk to third parties, considerations present but less explicitly operationalized in the US statements. Both frameworks also caution that they describe medical risk assessment rather than formal competition eligibility: in the United States, eligibility for interscholastic, collegiate, and professional competition is frequently determined separately by team, school, or league medical staff and governing bodies, while in Europe formal eligibility may be governed by national sports federations and international governing bodies, each of which may apply criteria that differ from, and in some cases are more restrictive than, the guideline recommendations reviewed here. Clinicians should treat this review, and the underlying HRS, AHA/ACC, and ESC documents, as decision support for medical risk assessment rather than a substitute for confirming an individual athlete’s formal eligibility with the relevant sporting authority.

Because sport-related terminology is used inconsistently across the literature, this review distinguishes recreational sport (undertaken primarily for personal enjoyment or fitness, without formal competition), competitive sport (organized participation with formal competition at any level), elite or professional sport (competition at the highest organized level, often with financial, contractual, or eligibility stakes), collision or contact sport (participation with expected or frequent direct bodily impact), and high-consequence sport (activities in which transient incapacitation carries a risk of death or catastrophic injury independent of the underlying arrhythmia, such as open-water swimming, scuba diving, climbing, or motorsport). These categories are not mutually exclusive, and an individual athlete’s risk is better characterized by the specific combination of physiologic demand, environment, and consequence of impairment than by any single label.

## 2. Evidence That Shifted the Field

Historically, sports eligibility recommendations for athletes with ICDs were conservative, largely because prospective data were limited and the consequences of device therapy or arrhythmic syncope during sport were uncertain [1,2,3,4]. The ICD Sports Safety Registry was therefore pivotal because it prospectively evaluated the outcomes that had driven these restrictions: death during sport, externally resuscitated tachyarrhythmia, shock-related injury, and failure of the device to terminate malignant ventricular arrhythmias [5]. Key studies informing contemporary sports participation recommendations for athletes with ICDs are summarized in Table 1.

In the original prospective, multinational registry, 372 athletes with ICDs, aged 10 to 60 years, were enrolled while participating in organized sports or activities considered high risk if a transient loss of control occurred [5]. Over a median follow-up of 31 months, there were no deaths, no resuscitated arrests, and no arrhythmia- or shock-related injuries during sports participation. ICD therapies were not rare: shocks occurred during competition or practice in 37 athletes (10% of the study population), during other physical activity in 29 athletes (8%), and at rest in 24 athletes (6%). Multiple shocks occurred during ventricular tachyarrhythmias, but the ICD ultimately terminated all episodes. Thus, the central message of the registry was not that vigorous sport eliminates arrhythmic risk, but that selected athletes could participate without the catastrophic outcomes that had historically driven broad restriction.

Longer-term and pediatric data reinforced this balanced message. In the extended follow-up of the ICD Sports Safety Registry, 440 athletes were followed for a median of 44 months, totaling 1446 person-years, again without sports-related death, externally resuscitated tachyarrhythmia, or serious arrhythmia- or shock-related injury [6]. In a subanalysis of 129 athletes aged 10 to 21 years, 35 athletes (27%) received 38 shocks, and one ventricular tachycardia (VT)/ventricular fibrillation (VF) storm occurred during competition, yet there were no deaths, arrests, or arrhythmia-related injuries during sports participation [7]. Lead malfunction-free survival was 92.3% at 5 years and 79.6% at 10 years, underscoring that long-term device durability remains important in young active patients [7]. The European cohort of intensive recreational athletes further broadened the relevance of these findings, emphasizing that counseling should be guided by physiologic demand and sport environment rather than by the label of “competitive” or “recreational” alone [8].

These data should be interpreted as reassuring but not definitive. Participants had a long time from implant to enrollment, many had already chosen to remain active despite their ICD, and many self-enrolled outside traditional recruitment pathways. This self-selection introduces a form of healthy-participant or survivor bias: athletes who continued competing after ICD implantation, and who agreed to enroll, are systematically different from those who stopped, were never referred, or declined participation. The underlying diseases and sports represented are also heterogeneous, and the number of athletes within any individual disease substrate is often small, limiting the precision of substrate-specific risk estimates. Collision sports, high-consequence sports, remote endurance activities, athletes with unstable arrhythmia profiles, and those with progressive cardiomyopathic substrates remain underrepresented or require cautious interpretation [5,6,7,8]. Taken together, this evidence base should be interpreted as reassuring for appropriately selected athletes rather than as demonstrating that unrestricted competitive sports participation is universally safe. In addition, much of the original prospective athlete data involved transvenous ICD (TV-ICD) systems and earlier programming strategies. A 2025 meta-analysis pooling these and related cohorts (six studies, 1183 athletes with TV-ICDs or S-ICDs) reported appropriate shock rates of 13% and inappropriate shock rates of 4%, with no shock-related physical injuries and a sports-discontinuation rate of only 2%, reinforcing the individual registries at the aggregate level while still relying on the same underlying observational, self-selected cohorts [11].

Newer device platforms further highlight the need for contemporary data. Subcutaneous ICDs (S-ICDs) may be attractive for young, active patients. Real-world data from the iSUSI registry suggest that S-ICDs can be used in athletes without an observed increase in overall device-related complications or inappropriate shocks compared with nonathletes, although myopotential oversensing and early post-implantation infections require attention [12]. Similarly, the multicenter S-ICD Rhythm Detect registry, which specifically stratified patients by self-reported physical activity level using a validated questionnaire, found no significant difference in arrhythmia- or device-related complications between inactive, moderately active, and highly active S-ICD recipients, further supporting the safety of an active lifestyle in appropriately selected S-ICD patients [17]. The ongoing SPORT S-ICD study was designed to prospectively evaluate sports participation among patients with S-ICDs [18]. Extravascular ICD (EV-ICD) systems and selected nontraditional approaches may expand options for athletes in whom avoiding intravascular leads is desirable, but athlete-specific outcome data remain limited [9,19].

## 3. Indications for ICD Implantation in Athletes

The decision to implant an ICD in an athlete should be driven by SCD risk rather than by the athlete’s desire to continue sport. Athletic participation may influence risk interpretation, counseling, device selection, programming, and follow-up, but it should not independently create an ICD indication. This distinction is particularly important in young athletes, for whom ICD implantation carries decades of potential shocks, generator changes, lead or system complications, body image concerns, eligibility implications, and psychological burden. Contemporary hypertrophic cardiomyopathy (HCM) guidance explicitly states that ICD placement solely to permit competitive athletics should not be performed [20].

ICD indications in athletes can be organized into three practical categories: secondary prevention, primary prevention, and gray areas. Secondary prevention indications are generally the most straightforward and include resuscitated SCA, sustained VT, VF, or hemodynamically significant ventricular arrhythmias not explained by a completely reversible cause [15]. Primary prevention decisions should be anchored in the underlying diagnosis and the quality of available risk markers. They are more nuanced and require substrate-specific risk assessment.

Gray areas arise when risk markers are incomplete, the phenotype is evolving, the event may have been partially reversible, or the athlete’s anticipated exercise exposure creates concern beyond what is captured by standard risk models. These most challenging scenarios are usually not clear indications. In these situations, clinicians should avoid using ICD implantation as a shortcut to permit sport participation or resolve uncertainty. Instead, the goal is to clarify the substrate, define the uncertainty, discuss the long-term consequences of device therapy, and separate the decision for sudden death prevention from the later decision about return to sport [9,15,20].

Maintaining this separation between diagnosis and desire preserves the integrity of sudden death prevention while allowing return-to-sport planning to focus on the factors most likely to reduce harm: substrate control, device selection, exercise-informed programming, emergency planning, and longitudinal reassessment.

## 4. Device Selection and Implantation Strategy in the Athlete

The optimal system depends on the athlete’s arrhythmic substrate, need for bradycardia pacing, ATP, cardiac resynchronization therapy (CRT), vascular access, anatomy, age, anticipated device longevity, sport type, and risk of trauma or repetitive mechanical stress. In athletes, the goal is not simply to implant a device capable of terminating VT or VF, but to select a system that provides durable protection while minimizing avoidable shocks, lead complications, pocket-related morbidity, and sport-specific limitations over years or decades of activity. A practical device-selection framework is summarized in Table 2.

The approach should begin by identifying clinical needs. Athletes who require chronic bradycardia pacing or cardiac resynchronization therapy require a transvenous system, since neither the S-ICD nor the EV-ICD provides these functions. Athletes who require ATP for monomorphic VT can be served by a transvenous ICD or, in selected cases, by an EV-ICD, which provides ATP and post-shock pacing but not chronic bradycardia support. In contrast, athletes without pacing needs who are young, have long-anticipated device exposure, or have a strong rationale for avoiding intravascular leads may be better suited for an S-ICD or EV-ICD. Recent case reports illustrate this individualized decision-making in practice, including exercise-guided S-ICD programming in a competitive athlete and EV-ICD implantation in a professional powerlifter, both without adverse effects on training or device function [13,14].

This decision process can be organized as a simple algorithm. The first branch point is pacing requirement: athletes with chronic bradycardia pacing or CRT indications require a transvenous system, since neither the S-ICD nor the EV-ICD provides these functions. Among athletes without a chronic pacing requirement, the second branch point is anticipated benefit from ATP: those with recurrent monomorphic VT likely to respond to ATP are candidates for a transvenous ICD or, in selected cases, an EV-ICD, while those without an ATP indication and with a strong rationale for avoiding intravascular leads are reasonable candidates for an S-ICD. Within each branch, the specific choice among transvenous, subcutaneous, and extravascular platforms is then refined by anatomy and vascular access, anticipated duration of device therapy, suitability of the athlete’s surface ECG for reliable sensing, sport-specific trauma exposure, and the athlete’s own preference after shared decision-making. Device-specific considerations are summarized in Table 3.

TV-ICDs remain the most versatile platform. The significant tradeoff is the long-term vulnerability of the transvenous lead. Young and active patients face cumulative exposure to lead fracture, insulation failure, venous obstruction, tricuspid valve interaction, infection, and extraction risk. In athletes participating in contact, collision, heavy resistance, or upper-body dominant sports, these risks should be discussed explicitly during device counseling and implantation planning [16].

S-ICDs are attractive for many athletes because they avoid the intravascular space and may reduce transvenous lead-related complications. In patients without pacing indications, randomized and prospective data support the safety and efficacy of S-ICD therapy [21,22,23]. However, the absence of bradycardia pacing, ATP, and CRT remains clinically important. S-ICDs may be less suitable for athletes with recurrent monomorphic VT, conduction disease, anticipated pacing needs, or substrates in which ATP could meaningfully reduce shocks.

S-ICD use in athletes also requires careful attention to sensing. Exercise may alter QRS amplitude, T-wave morphology, myopotential noise, respiratory pattern, and body position, all of which can affect sensing behavior. In athletic patients, S-ICD screening should ideally extend beyond resting vectors and include exercise testing, postural changes, and, when relevant, maneuvers that reproduce sport-specific myopotential noise or body position. This is especially important in athletes with high peak heart rates, potential T-wave changes during exertion, repetitive upper-extremity activity, prone positioning, trunk rotation, or direct chest wall contact.

Standard supine and upright screening at rest is an established, guideline-supported component of S-ICD candidacy assessment. Extending screening to exercise, posture changes, and sport-specific maneuvers is a reasonable, physiologically motivated extension of this practice, but it is supported predominantly by expert opinion and limited observational experience rather than by dedicated prospective trials and should be interpreted accordingly.

EV-ICD systems may offer an intermediate approach by avoiding intravascular leads while preserving selected pacing capabilities, including ATP and pause-prevention pacing. This concept is appealing for some athletes in whom transvenous lead avoidance is desirable but ATP or limited pacing support may still be clinically valuable [19,24]. However, these systems are newer than conventional TV-ICD and S-ICD platforms, and athlete-specific data remain limited. Their use should therefore be individualized.

Selected athletes may require nontraditional systems, including epicardial or hybrid approaches. These strategies are most relevant in complex congenital heart disease, limited venous access, intracardiac shunts, prior device infection, small body size, or anatomy that makes standard systems unfavorable. This may include an abdominal generator with epicardial pacing leads and pericardial or subcutaneous ICD coils, which could provide better protection from device harm.

Implantation strategy should account for the athlete’s sport before the procedure whenever possible. Pocket location, lead course, anchoring, equipment contact points, repetitive arm motion, and anticipated direct impact should be considered during preprocedural planning. For example, athletes in collision sports may be exposed to direct generator trauma; swimmers, rowers, climbers, baseball players, volleyball players, and weightlifters may place repetitive stress across the shoulder girdle; and cyclists, skiers, and runners may have sport-specific equipment or body positions that affect generator comfort. A lateral S-ICD generator may be well-tolerated in many patients but may be problematic in athletes exposed to direct thoracic impact, tight equipment, repetitive arm swinging, or prone positioning. Similarly, transvenous lead stress may be relevant in athletes with repetitive shoulder abduction, external rotation, heavy resistance training, falls, or collision exposure. These factors rarely determine device choice alone, but they should shape counseling, implantation approach, and follow-up.

The best device for an athlete is therefore not necessarily the newest or least invasive system, but the system that best matches the athlete’s arrhythmic risk, anatomy, sport exposure, and long-term management needs.

## 5. Device Programming in Athletes

Device programming is one of the most modifiable components of return-to-sport risk mitigation in athletes with ICDs. Athletes are vulnerable to inappropriate or avoidable therapies because exercise produces sustained sinus tachycardia, adrenergic surges, supraventricular arrhythmias, dynamic QRS and T-wave changes, myopotential noise, and body-position effects that may not be evident at rest. These factors can cause overlap between expected training heart rates and conventional detection zones, particularly in young athletes and those participating in high-intensity endurance sports. Therefore, ICD programming in athletes should be individualized rather than left at nominal or default settings.

The goal of programming is to reduce unnecessary therapies without compromising treatment of life-threatening VT. In broader ICD populations, high-rate detection thresholds, longer detection intervals, and modern discrimination algorithms reduce inappropriate or unnecessary therapies [25,26,27,28]. Athlete-specific data from the ICD Sports Registry support this approach directly: programming with higher rate cutoffs and longer detection intervals was associated with fewer inappropriate shocks and transient loss of consciousness without an increase in mortality [29]. These principles are highly relevant in athletes, but they must be applied in the context of the athlete’s substrate, prior arrhythmia history, and expected exercise physiology. An athlete with prior rapid VF may require a different programming strategy than an athlete with slower monomorphic VT that is consistently responsive to ATP. Similarly, athletes with frequent supraventricular tachycardia, atrial fibrillation, or sinus rates approaching standard therapy zones require careful discriminator programming and, when appropriate, treatment of the supraventricular arrhythmia substrate before return to sport. In practice, athlete-specific programming should aim to minimize inappropriate therapy from sinus tachycardia or supraventricular arrhythmias, reduce unnecessary therapy for nonsustained ventricular arrhythmias, preserve prompt treatment of malignant ventricular arrhythmias, confirm sensing during exertion, and prompt reassessment after a shock or meaningful clinical change.

Exercise testing is central to athlete-specific ICD programming. Maximal or sport-specific testing can define peak sinus rate, evaluate exertional ectopy, identify supraventricular arrhythmias, assess QRS and T-wave behavior, and reveal sensing abnormalities that are absent at rest [9]. When feasible, testing should approximate the athlete’s training conditions and then be used to determine appropriate detection zones and time to therapy.

In practice, this individualization can be organized as a sequential workflow. First, establish the athlete’s maximal or near-maximal physiologic sinus rate and typical training heart rate range through exercise or sport-specific testing. Second, document the rate and morphology of any previously recorded ventricular tachyarrhythmia, since therapy zones should be set with reference to the athlete’s own documented VT/VF rates rather than nominal defaults. Third, program detection and therapy zones with an adequate safety margin between the athlete’s peak sinus rate and the lowest programmed detection rate, using rhythm discrimination algorithms to further reduce inappropriate classification of supraventricular rhythms. Fourth, confirm adequate sensing during exercise testing, since myopotential noise, T-wave oversensing, and posture-dependent signal changes may only become apparent under exertional conditions. Fifth, interrogate the device after exercise testing and after any subsequent shock or clinical change to confirm that programming remains appropriate. This sequence does not require universal numerical thresholds, which are neither available nor appropriate given the heterogeneity of athletes and devices, but it does require that each step be performed deliberately rather than assumed.

Programming should be reassessed whenever the athlete’s clinical or training status changes. New symptoms, syncope, palpitations, appropriate or inappropriate ICD therapy, medication changes, catheter ablation, cardiomyopathy progression, or meaningful changes in training intensity should prompt device review. Remote monitoring can support early recognition of arrhythmia episodes, lead or sensing abnormalities, and therapy delivery, but it should not replace structured follow-up during return to high-intensity sport. Its ability to detect events earlier should not be equated with evidence that remote monitoring itself reduces sports-related adverse outcomes, which has not been directly demonstrated in athletes.

Programming deserves the same deliberate attention as device selection, not a settings checklist to finish and forget. The safest approach is iterative: define the athlete’s peak physiologic sinus rate, understand the arrhythmic substrate, program detection and therapy zones with appropriate safety margins, test the system under exertional conditions, and reassess after clinical or training changes. A practical approach to athlete-specific ICD programming is summarized in Table 4.

## 6. Return-to-Sport Risk Mitigation and Shared Decision-Making

Return-to-sport planning works best as an ongoing, structured process; clearance is a starting point, not a single yes-or-no verdict. This process should be individualized because the same arrhythmic substrate may carry different practical implications in a supervised training facility, collegiate field, open water, remote terrain, or collision sport. Return-to-sport assessment should occur only after disease-specific therapy has been optimized, including trigger avoidance, hydration, pharmacotherapy, and left cardiac sympathetic denervation when indicated.

Emergency preparedness is the foundation of risk mitigation. The presence of an ICD should not lead athletes, families, teams, or clinicians to assume that external emergency planning is unnecessary. ICD therapies may fail, syncope may occur before therapy is delivered, and non-arrhythmic emergencies can still arise during sport. A venue-specific emergency action plan should include rapid recognition of SCA, activation of emergency medical services, immediate cardiopulmonary resuscitation, access to an automated external defibrillator, trained responders, and a clear process for transfer to medical care [9,30,31]. For athletes training outside organized settings, these principles should be adapted to the environment, including training partners, communication access, route planning, and proximity to emergency services.

Sport modifies risk through at least three distinct mechanisms, which should be considered separately rather than conflated. First, exercise-related arrhythmic or substrate risk reflects the effect of exertion itself on the underlying arrhythmic substrate, through catecholaminergic surge, hemodynamic load, or, in some cardiomyopathies, disease progression. Second, mechanical or device trauma risk reflects the potential for direct impact, compression, or repetitive strain that could damage the generator or leads, independent of arrhythmic risk. Third, consequence-of-incapacitation risk reflects the setting in which the athlete competes: a transient loss of consciousness or brief impairment that would be inconsequential on a track may be catastrophic during open-water swimming, solo climbing, motorsport, or other high-consequence environments, regardless of how promptly the ICD terminates the arrhythmia. Collision sports predominantly raise concerns related to the second mechanism, whereas high-consequence sports are defined primarily by the third; a comprehensive risk assessment should evaluate all three mechanisms independently rather than assuming that addressing arrhythmic risk alone is sufficient.

Sport-specific hazards should be discussed explicitly. High-consequence activities are those in which transient loss of consciousness, presyncope, or shock could cause harm independent of the arrhythmia itself, such as open-water swimming, scuba diving, climbing, motorsport, cycling in traffic, downhill skiing, solo endurance events, and activities performed in remote or altitude environments. Collision and contact sports raise separate concerns related to direct generator trauma, lead stress, pocket pain, or equipment-related compression. These activities are not interchangeable, and risk is not automatically comparable across them: some sports, such as unaccompanied open-water swimming, scuba diving, or solo climbing, carry a real risk of death from even brief impairment and require separate, sport-specific assessment; participation may remain inadvisable in some cases despite otherwise acceptable arrhythmic risk. As a general principle, these risks do not automatically preclude participation, but they should be named directly and incorporated into the return-to-sport plan.

A post-shock plan should be established before the athlete returns to sport. Athletes should know when to stop activity, when to seek urgent medical evaluation, and when device interrogation is required. In general, exercise should be stopped after ICD therapy until the episode is reviewed, the rhythm diagnosis is confirmed, reversible triggers are assessed, device function is evaluated, and programming or medical therapy is adjusted when needed. Return to sport after an ICD therapy should be individualized and should not occur simply because symptoms have resolved.

The absence of sports-related mortality or catastrophic injury in the major registries is reassuring, but ICD therapies in these cohorts were not uncommon, and the psychological consequences of appropriate and inappropriate shocks deserve explicit attention. Fear of shock recurrence, hypervigilance, and avoidance behavior can affect training, competition, and quality of life independent of any residual arrhythmic risk and should be assessed alongside physical readiness when planning return to sport.

Contemporary international statements provide several operational benchmarks. The 2024 HRS Expert Consensus Statement on Arrhythmias in the Athlete considers return to sport reasonable 4–6 weeks after a new ICD implantation and 2 weeks after generator replacement [9], whereas the 2025 AHA/ACC scientific statement recommends restricting sports participation for 4–8 weeks after implantation and for 2 weeks after generator replacement [10]. In athletes returning after sudden cardiac arrest, the HRS consensus also recommends maximum-effort exercise testing to assess exertional arrhythmias, peak heart rate, and appropriate ICD sensing, as well as programming with long detection durations and high-rate cutoffs to reduce unnecessary therapies [9]. When feasible, exercise testing should reproduce the physiological demands of the athlete’s sport [9]. After an ICD shock, return to sport should be deferred until the cause of the arrhythmia has been identified, the underlying condition addressed, and appropriate device function confirmed [9,10].

Shared decision-making is the clinical process that integrates these risks with the athlete’s goals and values. It should be a structured conversation in which the clinician explains known risks, acknowledges uncertainty, reviews reasonable alternatives, and documents the agreed-upon mitigation strategy. Relevant stakeholders may include family members, coaches, athletic trainers, school officials, team physicians, and emergency responders, particularly for pediatric, collegiate, elite, or professional athletes. Clinicians should also recognize when external pressures from teams, institutions, or family members may conflict with the athlete’s best interest.

**Figure 1:**
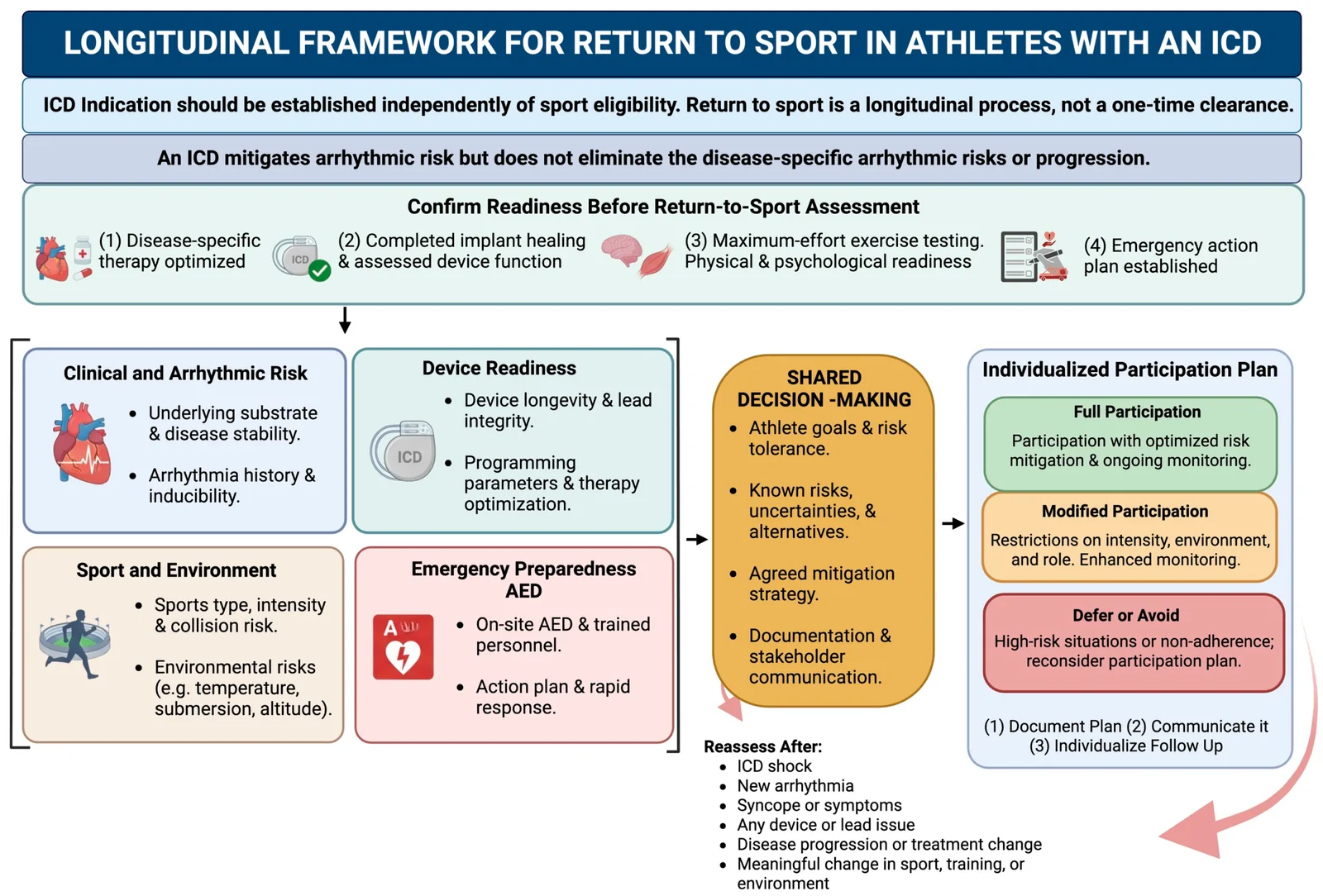
Longitudinal framework for return to sport in athletes with an implantable cardioverter-defibrillator. The indication for an implantable cardioverter-defibrillator (ICD) should be established independently of sport eligibility. Before return-to-sport assessment, disease-specific therapy should be optimized, implant healing and device function confirmed, maximum-effort exercise testing completed, and physical and psychological readiness established. Evaluation integrates clinical and arrhythmic risk, device readiness, sport- and environment-specific hazards, and emergency preparedness. These domains inform shared decision-making that incorporates the athlete’s goals and risk tolerance, known risks and uncertainties, available alternatives, risk-mitigation strategies, and stakeholder communication. The resulting individualized plan may support full participation, modified participation, or deferral. An ICD shock, new arrhythmia, syncope or other symptoms, device or lead issue, disease progression or treatment change, or meaningful change in sport, training, or environment should prompt reassessment. Return to sport is therefore a longitudinal process rather than a one-time clearance. AED = automated external defibrillator; ICD = implantable cardioverter-defibrillator.

A practical algorithm for return-to-sport risk mitigation and shared decision-making is presented in Figure 1.

## 7. Special Situations

Several clinical scenarios require additional caution when applying a return-to-sport approach to athletes with ICDs. These situations do not necessarily mandate restriction, but they often require more granular counseling, closer follow-up, and clearer boundaries around participation.

Risk differs substantially by underlying disease. In arrhythmogenic cardiomyopathy (ACM), exercise itself may accelerate disease progression and worsen the arrhythmic substrate, so the presence of an ICD does not remove the rationale for exercise restriction that exists independent of shock risk. Genotype-specific risk further refines this assessment: pathogenic variants in LMNA, FLNC, RBM20, PLN, and desmosomal genes are associated with disproportionately high arrhythmic risk that may not be fully captured by conventional phenotypic markers such as left ventricular ejection fraction, and the 2023 ESC cardiomyopathy guidelines incorporate genotype into contemporary risk stratification and ICD decision-making [32,33]. Brugada syndrome carries its own trigger-specific considerations, including fever and certain pharmacologic exposures, that are independent of exercise per se but warrant the same disease-specific counseling applied to other channelopathies. In catecholaminergic polymorphic ventricular tachycardia (CPVT) and long QT syndrome (LQTS), risk is closely tied to adrenergic state and trigger avoidance, and beta-blocker adherence and trigger-specific counseling remain central regardless of device therapy [9,10,34]. In hypertrophic cardiomyopathy (HCM), outflow tract dynamics and exertional hemodynamics inform risk beyond arrhythmia alone. Myocarditis carries a time-limited, higher risk window tied to active inflammation, though long-term fibrosis may occur. Congenital heart disease introduces anatomic and hemodynamic considerations that are independent of the ICD itself. These differences mean that return-to-sport planning must be disease-specific rather than device-specific, with the ICD treated as one component of a substrate-driven risk assessment rather than a substitute for it.

Pediatric and adolescent athletes require particular attention because return-to-sport decisions occur within the context of family dynamics, school policies, developmental stage, peer identity, and long-anticipated device exposure. Pediatric registry data are reassuring in that selected young athletes with ICDs have participated in sports without observed deaths, arrests, or arrhythmia-related injuries during sports participation; however, shocks and lead malfunction remain clinically important over time [7]. Counseling should include the athlete and family and address school emergency action plans, athletic trainer availability, medication adherence, psychosocial support, and the likelihood of future lead or generator interventions.

Elite and professional athletes present a different set of challenges. Their training intensity, public visibility, financial incentives, and external pressure to return may be substantially greater than those of recreational athletes. These factors can distort risk tolerance and complicate shared decision-making. Clinicians should distinguish the athlete’s own goals from expectations imposed by teams, sponsors, institutions, or family members. Documentation should be especially clear regarding the residual risks discussed, alternatives considered, emergency planning, and the athlete’s voluntary preference.

A 2026 retrospective analysis of sporting careers after ICD implantation in elite athletes offers descriptive insight into this population, though its media- and literature-based case identification carries substantial selection and ascertainment bias and should be interpreted as hypothesis-generating rather than definitive [35].

Return to sport after ICD therapy should be handled deliberately. An appropriate shock may indicate recurrent ventricular arrhythmia, progression of the underlying substrate, medication nonadherence, electrolyte disturbance, excessive exertional stress, or the need for ablation or medication adjustment. An inappropriate shock may reflect sinus tachycardia, supraventricular arrhythmia, oversensing, lead or sensing abnormalities, or programming issues. In either case, return to sport should generally be deferred until stored electrograms have been reviewed, the rhythm diagnosis has been confirmed, reversible triggers have been addressed, device function and programming have been reassessed, and the athlete’s emergency action plan has been updated.

These special situations are not exceptions to the return-to-sport framework. They are scenarios in which the structure must be applied with greater specificity. The central question remains whether the athlete can participate with an acceptable residual risk after the substrate, device system, programming strategy, sport environment, emergency plan, and follow-up structure have been deliberately addressed.

## 8. Future Directions

Future research should move beyond the broad question of whether selected athletes with ICDs can participate in sport. The next phase of evidence should define which athletes can participate, in which sports, with which device systems, under which programming strategies, and with what emergency-planning infrastructure. The original ICD Sports Registry established the foundation for contemporary return-to-sport counseling, but modern practice has evolved substantially. Athletes are now considered for lead-sparing systems, programmed with contemporary shock-reduction strategies, managed through remote monitoring, and counseled within a shared decision-making framework that must account for sport-specific hazards and patient values.

Contemporary data are particularly needed for lead-sparing ICD systems. S-ICDs are attractive for many young and active patients because they avoid transvenous leads, yet most prospective athlete data were generated in transvenous ICD populations. Future studies, such as the SPORT S-ICD study, should determine how S-ICD sensing performs during high-intensity exercise, repetitive upper-extremity activity, posture changes, direct impact, and sport-specific body positions [18]. The ongoing ORCCA registry also includes athletes with ICDs that may inform the discussion [36].

EV-ICD systems represent another important research priority. These systems may offer a potential middle ground between transvenous and subcutaneous platforms by avoiding intravascular leads while preserving selected pacing capabilities, including ATP. Long-term pivotal data support EV-ICD performance and safety in broader ICD populations, but athlete-specific outcomes remain limited [24].

Future registries should also deliberately include populations and sports that remain underrepresented. These include pediatric and adolescent athletes, female athletes, elite and professional athletes, athletes with adult congenital heart disease [37], athletes with arrhythmogenic cardiomyopathy [34], and athletes participating in collision, water, motor, remote, or other high-consequence sports. These groups are clinically important because risk is shaped not only by arrhythmia probability, but also by developmental stage, anatomy, disease progression, external pressure to participate, device durability, and the consequences of transient impairment in a specific environment.

## 9. Conclusions

The presence of an ICD should no longer be viewed as an automatic exclusion from vigorous or competitive sport. Contemporary registry data and expert consensus support an individualized approach in which selected athletes with ICDs may participate when the underlying substrate, device system, programming strategy, sport environment, emergency planning, and follow-up structure are deliberately addressed [5,6,7,8,9]. However, these data should not be interpreted as an absence of risk. ICD therapies occur, inappropriate shocks remain clinically relevant, device durability matters, and some sport environments carry consequences that extend beyond the arrhythmia itself.

The modern care of athletes with ICDs, therefore, requires more than a clearance decision. The decision to implant an ICD stays anchored to the underlying arrhythmic diagnosis, independent of any athletic ambition. Device selection should account for pacing needs, ATP, anatomy, age, sport exposure, and long-term hardware considerations. Programming should be informed by exercise physiology and reassessed after shocks, symptoms, medication changes, ablation, or changes in training intensity. Risk mitigation should include emergency action planning, access to automated external defibrillators, post-shock protocols, trauma prevention, remote monitoring, and structured follow-up.

Shared decision-making sits at the center of this process, but it only works if it stays rigorous, a real conversation about risk rather than a formality on the way to yes. Clinicians should communicate known risks, acknowledge uncertainty, document the athlete’s goals and values, and define practical steps to reduce modifiable hazards. The goal is not to make sport risk-free, but to help selected athletes participate with the lowest achievable and most transparent residual risk.

## Figures and Tables

**Table 1:** Key evidence informing sports participation in athletes with implantable cardioverter-defibrillators.

Study	Population/Design	Key Findings	Limitations	Practical Implication
Lampert et al., ICD Sports Safety Registry, 2013 [5] ^†^	Prospective multinational registry of 372 athletes with ICDs, aged 10–60 years, participating in organized sports or high-risk activities; median follow-up 31 months	No deaths, resuscitated arrests, or arrhythmia- or shock-related injuries during sports participation. Shocks occurred during competition/practice in 37 athletes, during other physical activity in 29, and at rest in 24. All ventricular tachyarrhythmia episodes were ultimately terminated by the ICD.	Selected cohort; participants had already chosen to remain active; limited representation of some collision, remote, and high-consequence sports.	Pivotal evidence supporting movement away from automatic sports disqualification in selected athletes with ICDs.
Lampert et al., long-term ICD Sports Registry follow-up, 2017 [6] ^†^	Extended follow-up of 440 athletes with ICDs; median follow-up 44 months, totaling 1446 person-years	No sports-related deaths, externally resuscitated tachyarrhythmias, or serious arrhythmia- or shock-related injuries.	Registry design and selection bias; findings may not apply to all substrates, sports, or unstable arrhythmia profiles.	Reinforced the durability of the original registry findings and supported longitudinal return-to-sport management.
Saarel et al., pediatric and young athlete ICD Sports Registry analysis, 2018 [7] ^†^	Subanalysis of 129 athletes aged 10–21 years with ICDs	Thirty-five athletes received 38 shocks; one VT/VF storm occurred during competition. No deaths, arrests, or arrhythmia-related injuries occurred during sports participation. Lead malfunction-free survival was 92.3% at 5 years and 79.6% at 10 years.	Pediatric decisions involve family, school, psychosocial, and long-term device considerations; lead durability remains important.	Supports selected youth sports participation while emphasizing shock preparedness, school-based EAPs, and long-term hardware surveillance.
Heidbuchel et al., European cohort of the ICD Sports Safety Registry, 2019 [8] ^†^	European cohort of intensive recreational athletes with ICDs	Intensive recreational athletes may experience physiologic demands similar to competitive athletes. Counseling should consider training intensity and sport environment, not only competition status.	Recreational athletes are heterogeneous; findings may not apply to all high-consequence or collision sports.	Encourages counseling based on physiologic demand, sport setting, and emergency access rather than the label of “competitive” versus “recreational.”
Link et al., ICD lead survival in athletic patients, 2021 [16]	Study evaluating ICD lead survival in athletic patients	Lead malfunction and hardware complications remain important considerations in active patients, particularly over long follow-up.	Lead risk varies by age, device type, sport mechanics, and follow-up duration.	Device durability should be part of counseling, especially for young athletes and those in contact, collision, resistance, or upper-body dominant sports.
Gasperetti et al., iSUSI registry, 2025 [12]	Multicenter, real-world International Subcutaneous ICD (iSUSI) registry; 1493 total S-ICD recipients, of whom 152 (10.2%) were athletes; median follow-up 25.5 months	No observed increase in overall device-related complications or inappropriate shocks in athletes versus nonathletes after adjustment for confounders. Appropriate shocks were more frequent in athletes and occurred more often during exercise; myopotential oversensing and early postimplantation infection required attention.	Retrospective observational registry; sports exposure was heterogeneous; uncommon sport-specific events may have been underpowered; and residual confounding remains possible.	Reassuring real-world evidence supporting S-ICD use in athletes, but findings should be interpreted cautiously given selection bias and retrospective design.
Francia et al., S-ICD Rhythm Detect registry, 2025 [17]	Multicenter registry of young S-ICD recipients stratified by self-reported physical activity level using a validated questionnaire	No significant difference in arrhythmia- or device-related complications between inactive, moderately active, and highly active patients.	Self-reported activity level; retrospective design; young-patient cohort may not generalize to all age groups.	Further supports the safety of an active lifestyle in appropriately selected S-ICD recipients across a range of physical activity levels.

Selected studies that informed the transition from categorical sports restriction toward individualized return-to-sport assessment in athletes with ICDs. Findings should be interpreted in the context of each study’s population, design, and limitations. Abbreviations: EAP, emergency action plan; ICD, implantable cardioverter-defibrillator; iSUSI, International Subcutaneous Implantable Cardioverter-Defibrillator Registry; S-ICD, subcutaneous implantable cardioverter-defibrillator; VF, ventricular fibrillation; VT, ventricular tachycardia. ^†^ these four analyses report serial or overlapping follow-up of the same underlying ICD Sports Safety Registry population and should not be interpreted as independent cohorts.

**Table 2:** Device selection and implantation considerations for athletes with implantable cardioverter-defibrillators.

Type of Device	Preferred Scenarios	Situations Requiring Caution	Athlete-Specific Implantation and Follow-Up Considerations	Practical Implication
Transvenous ICD	Athletes who require bradycardia pacing, antitachycardia pacing (ATP), or cardiac resynchronization therapy. This strategy is particularly relevant when recurrent monomorphic ventricular tachycardia is anticipated and may respond to ATP.	Young athletes with long anticipated device exposure; limited venous access; intracardiac shunt; elevated infection risk; or participation in contact, collision, heavy resistance, or upper-body dominant sports.	Preimplant counseling should address long-term lead durability, venous obstruction, tricuspid valve interaction, infection, extraction risk, and the potential effects of repetitive shoulder motion, direct trauma, falls, or heavy upper-body loading on lead and pocket integrity.	The transvenous ICD remains the most versatile platform, but its long-term intravascular lead burden is the principal tradeoff in young and highly active patients.
Subcutaneous ICD	Athletes with an ICD indication who do not require bradycardia pacing, ATP, or cardiac resynchronization therapy, particularly when preservation of venous access or avoidance of transvenous leads is prioritized.	Recurrent monomorphic ventricular tachycardia likely to benefit from ATP; sinus node dysfunction; atrioventricular conduction disease; current or anticipated pacing requirement; cardiac resynchronization indication; abnormal sensing vectors; or substantial exertional electrocardiographic changes.	Screening should include standard resting vector assessment and, when feasible, exercise testing, postural assessment, and maneuvers that reproduce sport-specific body position or myopotential noise. Generator location should be considered in relation to body habitus, equipment, comfort, and risk of direct impact.	The subcutaneous ICD is an attractive lead-sparing option for selected athletes, but its lack of ATP and pacing requires careful patient selection and deliberate sensing evaluation.
Extravascular ICD	Selected athletes in whom avoidance of intravascular leads is desirable but limited pacing capability, or ATP, may be clinically valuable. This strategy may be considered when an S-ICD is appealing, but lack of ATP is a significant limitation.	Limited operator experience; unfavorable anatomy or body habitus; concern for substernal lead placement; or clinical scenarios in which limited athlete-specific outcome data make counseling uncertain.	Evaluation should include expected ventricular tachycardia mechanism, likelihood of ATP benefit, pacing needs, chest anatomy, sport-related trauma exposure, and local expertise with implantation and longitudinal device management.	The extravascular ICD may represent an intermediate strategy between transvenous and subcutaneous systems, but its role in athletes remains evolving and should be individualized.
Epicardial or hybrid ICD systems	Athletes with complex congenital heart disease, limited venous access, intracardiac shunts, prior device infection, small body size, or anatomy that makes standard transvenous or subcutaneous systems unfavorable.	Patients in whom standard ICD systems are feasible and the added procedural complexity of a surgical or hybrid system is not justified.	Relevant considerations include venous anatomy, intracardiac shunting, thromboembolic risk, ventricular morphology, pacing requirements, prior surgical repairs, and scar-related ventricular tachycardia mechanisms.	These systems should be reserved for highly selected patients in whom anatomy, access, or substrate-specific factors drive device strategy.

Device selection should be individualized according to the underlying diagnosis and arrhythmia substrate, pacing and antitachycardia pacing requirements, anatomy and venous access, anticipated duration of device therapy, sport-specific mechanical exposure, and patient preferences. No single device platform is optimal for every athlete. Abbreviations: ATP, antitachycardia pacing; ICD, implantable cardioverter-defibrillator; S-ICD, subcutaneous implantable cardioverter-defibrillator.

**Table 3:** Quantitative comparison of transvenous, subcutaneous, and extravascular ICD platforms.

Characteristic	Transvenous ICD	Subcutaneous ICD	Extravascular ICD
**Antitachycardia pacing (ATP)**	Yes	No	Yes, though athlete-specific long-term data remain limited.
**Chronic Bradycardia Pacing/CRT**	Yes	No	No
**Sensing**	Intracardiac electrogram; well validated across activity levels.	Surface-vector sensing; requires dedicated screening and is more susceptible to myopotential noise and T-wave oversensing during exertion.	Surface-vector sensing similar in principle to S-ICD; athlete-specific sensing data remain limited.
**Lead/Generator Location**	Transvenous lead; pectoral generator; intravascular.	Subcutaneous parasternal lead; lateral thoracic generator; no intravascular component.	Substernal lead; lateral thoracic generator; no intravascular component.
**Mechanical/Trauma Exposure**	Lead fracture or dislodgement risk with repetitive motion or direct trauma; long-term lead durability is the principal concern.	Generator and lead exposed to direct impact and equipment compression in collision or contact sports; no intravascular lead fatigue risk.	Extrathoracic trauma exposure similar to S-ICD; substernal position may theoretically reduce some risks, but comparative data are limited.
**Athlete-Specific Evidence**	Most extensive (ICD Sports Registry and related analyses spanning over a decade).	Growing (iSUSI registry, case reports).	Limited data (isolated case reports only).

ATP, antitachycardia pacing; CRT, cardiac resynchronization therapy; ICD, implantable cardioverter-defibrillator; iSUSI, International Subcutaneous Implantable Cardioverter-Defibrillator Registry. Abbreviations; ICD, implantable cardioverter-defibrillator; S-ICD, subcutaneous implantable cardioverter-defibrillator.

**Table 4:** Athlete-specific implantable cardioverter-defibrillator programming and follow-up considerations.

Programming	Athlete-Specific Concern	Programming Strategy	Clinical Objective
Detection Rate Thresholds	Peak sinus rates during training may overlap with conventional ventricular tachycardia detection zones, particularly in young athletes and endurance athletes.	Use maximal or sport-specific exercise testing to define peak sinus rate. When clinically appropriate, program therapy zones above expected exercise sinus rates and consider monitor-only zones for diagnostic information.	Reduce inappropriate therapy from sinus tachycardia while preserving detection of clinically meaningful ventricular arrhythmias.
Detection Duration	Transient rate acceleration and nonsustained ventricular arrhythmias may occur during exertion.	Consider longer detection intervals when appropriate, particularly in athletes without prior unstable rapid ventricular arrhythmias.	Reduce unnecessary antitachycardia pacing and shocks for nonsustained or self-terminating arrhythmias.
Rhythm Discrimination	Sinus tachycardia, supraventricular tachycardia, and atrial fibrillation may mimic ventricular tachycardia during high-intensity exercise.	Ensure discriminators are active and appropriate for the device type, chamber configuration, and clinical substrate. Treat recurrent supraventricular arrhythmias when they increase the risk of inappropriate therapy.	Improve distinction between ventricular and supraventricular rhythms during exertion.
Antitachycardia Pacing (ATP)	Organized monomorphic ventricular tachycardia may be terminated without shock in transvenous or extravascular systems.	Program antitachycardia pacing when prior or anticipated ventricular tachycardia mechanism, rate, and hemodynamic tolerance support benefit. Avoid assuming antitachycardia pacing is appropriate for all substrates.	Reduce shocks in selected athletes while maintaining effective treatment of ventricular tachyarrhythmias.
Subcutaneous ICD Sensing	Exercise may alter QRS amplitude, T-wave morphology, posture, respiratory pattern, and myopotential noise, increasing the risk of oversensing.	Perform standard resting vector screening and, when feasible, exercise- or sport-specific screening with postural changes and maneuvers that reproduce sport conditions.	Identify exertion-related sensing concerns before return to high-intensity sport.
Post-Shock Evaluation	Appropriate or inappropriate shocks may recur if the underlying trigger, arrhythmia substrate, or programming issue is not addressed.	After any ICD therapy, review stored electrograms, confirm the rhythm diagnosis, assess reversible triggers, evaluate device function, and adjust programming when needed.	Prevent premature return to sport after an unresolved arrhythmia, sensing, lead, or programming problem.
Remote monitoring and Follow-up	Frequent training increases exposure to arrhythmias, sensing issues, and therapy delivery.	Use remote monitoring for early episode review, paired with structured follow-up during early return to sport, after shocks, and after meaningful changes in training intensity or medications.	Support rapid detection of clinically important events while maintaining deliberate longitudinal oversight.

Programming should be individualized according to the underlying diagnosis, arrhythmia history, device type, measured peak exercise heart rate, and anticipated sport-specific physiological demands. The strategies presented are general considerations and should not be interpreted as universal programming parameters. Device therapy or a clinically meaningful change in symptoms, training, medications, or disease status should prompt reassessment. Abbreviations: ATP, antitachycardia pacing; ICD, implantable cardioverter-defibrillator; QRS, QRS complex.

## Data Availability

No new data were created or analyzed in this study. Data sharing is not applicable to this article.

## Abbreviations

AED	Automated external defibrillator
ATP	Antitachycardia pacing
CRT	Cardiac resynchronization therapy
EAP	Emergency action plan
EV-ICD	Extravascular implantable cardioverter-defibrillator
HCM	Hypertrophic cardiomyopathy
ICD	Implantable cardioverter-defibrillator
SCA	Sudden cardiac arrest
SCD	Sudden cardiac death
SDM	Shared decision-making
S-ICD	Subcutaneous implantable cardioverter-defibrillator
TV-ICD	Transvenous implantable cardioverter-defibrillator
VF	Ventricular fibrillation
VT	Ventricular tachycardia
